# Pleurisy and Empyema in Children

**Published:** 1884-01

**Authors:** 


### Pleurisy and Empyema in Children.

In giving the symptoms and physical signs of pleuritis, as exemplified in a patient twenty months old, Dr. Pepper called attention to the presence of bronchial breathing in the affected side, and to the making of the characteristic symptoms in the early stage of the attack.

The common statement is, that over large pleuritic effusions there is, in addition to the flatness or percussion, feebleness or absence of respiratory murmur. The vesicular murmur is, in truth, usually suppressed under such circumstances, but it is not uncommon to hear bronchial breathing. This has certain peculiarities in pleuritis, viz., it seems distant, and the respiratory element is most marked; it is, in fact, an exact counterpart of the laryngeal sound caused by obstructed breathing and disappears when the patient breathes with the mouth open so as to make no noise in the throat. The probability is that the sound is transmitted from a partially obstructed bronchial tube, from the larynx or trachea through the tense thoracic walls to the ear. Such bronchial breathing differs from that heard over a solidified lung, in that the latter seems immediately under the ear, and wants the great accentuation of the expiratory element.

In regard to the second point, the symptoms of acute pleuritis in children are often masked by the symptoms of disorder of the stomach or bowels, or by nervous symptoms simulating the onset of a specific fever. The pain, when the child is old enough to complain of it, is often referred not to the chest, but to the stomach, the back, or even the thigh. This difficulty in diagnosis is only to be overcome by invariably examining children thoroughly—especially those who are too young to tell their symptoms—and attending particularly to the heart and lungs.

The treatment advocated for the early stage is the application of leeches and counter-irritants, and later, when effusion has taken place, of blisters. Quinine must also be given in large doses with the object of counteracting the tendency, so marked in children, to the conversion of the serous effusion into pus. When fever has subsided, iodide and acetate of potassium may be administered, with digitalis if there is cardiac irritability.

If the effusion does not quickly disappear under this plan of management, an exploring puncture with a hypodermic needle should be made to determine the character of the fluid. If pus be found it should be removed by aspiration. Sometimes, more frequently than in adults, there is no re-accumulation, but if there should be, a silver tube with a valve or a flexible drainage tube must be inserted. Disinfecting injections are not often called for in children.

When left to nature the most favorable termination of empyema is spontaneous perforation of, and evacuation through, the chest walls. This occurs much more frequently in children than in adults. The point of perforation is usually on the antero-lateral surface of the chest, in the fourth interspace. The next most favorable termination is perforation into a bronchial tube. In both cases ultimate recovery is the usual result.

On the other hand hectic fever may develop and the child die exhausted or be carried off by some intercurrent disease of the stomach or bowels; or some latent tuberculous tendency may be developed; or finally the pus may concrete into a semi-solid mass and a lingering illness, resembling a slow phthisis, ensue. —*Philadelphia Medical Times.*

